# A Biotin Biosynthesis Gene Restricted to *Helicobacter*

**DOI:** 10.1038/srep21162

**Published:** 2016-02-12

**Authors:** Hongkai Bi, Lei Zhu, Jia Jia, John E. Cronan

**Affiliations:** 1 Department of Pathogenic Biology, Nanjing Medical University, 140 Hanzhong Road, Nanjing, Jiangsu 210029, China; 2 Key Laboratory of Pathogen Biology of Jiangsu Province, Nanjing Medical University, 140 Hanzhong Road, Nanjing, Jiangsu 210029, China; 3 Department of Microbiology, University of Illinois, 601 S. Goodwin Ave, Urbana, Illinois 61801, USA; 4 Department of Biochemistry, University of Illinois, B103 Chemical and Life Sciences Laboratory, 601 S. Goodwin Ave, Urbana, Illinois 61801, USA

## Abstract

In most bacteria the last step in synthesis of the pimelate moiety of biotin is cleavage of the ester bond of pimeloyl-acyl carrier protein (ACP) methyl ester. The paradigm cleavage enzyme is *Escherichia coli* BioH which together with the BioC methyltransferase allows synthesis of the pimelate moiety by a modified fatty acid biosynthetic pathway. Analyses of the extant bacterial genomes showed that *bioH* is absent from many *bioC-*containing bacteria and is replaced by other genes. *Helicobacter pylori* lacks a gene encoding a homologue of the known pimeloyl-ACP methyl ester cleavage enzymes suggesting that it encodes a novel enzyme that cleaves this intermediate. We isolated the *H. pylori* gene encoding this enzyme, *bioV*, by complementation of an *E. coli bioH* deletion strain. Purified BioV cleaved the physiological substrate, pimeloyl-ACP methyl ester to pimeloyl-ACP by use of a catalytic triad, each member of which was essential for activity. The role of BioV in biotin biosynthesis was demonstrated using a reconstituted *in vitro* desthiobiotin synthesis system. BioV homologues seem the sole pimeloyl-ACP methyl ester esterase present in the *Helicobacter* species and their occurrence only in *H. pylori* and close relatives provide a target for development of drugs to specifically treat *Helicobacter* infections.

*Helicobacter pylori* is the first formally recognised bacterial carcinogen and is an extremely successful human pathogen. Most of the world population harbor this gram-negative bacterium in their upper gastrointestinal tract. *H. pylori* causes gastritis and peptic ulcer disease, and is associated with lymphoma of gastric mucosa-associated lymphoid tissue and certain types of gastric cancer^1^. Following diagnosis the current approach is a combination of antibiotic and anti-secretory therapy followed 4 to 6 weeks later by testing that the infection has been cured^1^,^2^. Increased antibiotic resistance is largely responsible for failure of this treatment. The prevalence of cases resistant to the three main drugs (clarithromycin, metronidazole, and levofloxacin) is very high in many regions of the world^1^,^3^. Thus, development of new drugs effective against resistant strains has become a priority.

Biotin (vitamin H) is a key protein-bound enzyme cofactor required in all three domains of life^4^. Biotin functions as a covalently bound prosthetic group that plays essential roles in the transfer of CO_2_ in key central metabolic processes involving carboxylation, decarboxylation or transcarboxylation reactions^5^,^6^. Recently biotin has been proposed to be a critical and limited nutrient during infections by human pathogens such as *Mycobacterium tuberculosis*^7^ and *Francisella tularensis*^8^. Although biotin was discovered over 100 years ago, until recently it was not known how the pimelate moiety is synthesized^5^,^9^. Labelling studies in *Escherichia coli* suggested that most of the carbon atoms of biotin are derived from pimelic acid, a seven carbon α,ω-dicarboxylic acid^10^,^11^. The early steps for the pimelate moiety synthesis in *E. coli* were long an enigma until recent work by Lin and coworkers showed that two enzymes, BioC and BioH, hijack a fraction of the fatty acid biosynthetic capacity to make the pimelate moiety^9^,^12^. BioC is an *O*-methyltransferase that methylates the ω-carboxyl group of malonyl-acyl carrier protein (ACP) to form malonyl-ACP methyl ester, a molecule that is accepted as a primer by the fatty acid synthetic pathway^13^. The methyl ester is processed via two reiterations of the fatty acid elongation cycle to give a pimeloyl-ACP methyl ester, which is cleaved by BioH to give pimeloyl-ACP (Fig. 1a). Although BioH is a somewhat promiscuous carboxylesterase^9^,^14^, it was demonstrated that pimeloyl-ACP methyl ester is the physiological substrate of BioH^15^. Thus, BioH provides the gatekeeping function that blocks further elongation of this substrate by the fatty acid biosynthetic enzymes^15^. However, the enzymes that have BioH-like activity show marked sequence diversity among *bioC*-containing bacteria^16^. Three other enzymes, BioG, BioK^17^ and BioJ^18^, have been identified in bacteria that encode BioC but not BioH. Each of these enzymes has been shown to catalyse the cleavage of pimeloyl-ACP methyl ester *in vitro*^17^,^18^.

To our knowledge biotin synthesis in *H. pylori* has not been studied. This bacterium clearly makes biotin because biotin supplementation has no effect on growth in a chemically defined media^19^,^20^. Moreover, the essential biotinylated protein subunit of acetyl-CoA carboxylase, the first enzyme of fatty acid biosynthesis, has been demonstrated in *H. pylori* and the kinetics of the enzyme activity determined^21^. Analyses of the available *H. pylori* genomes showed that *H. pylori* encodes all of the proteins required for assembly of the fused heterocyclic rings of biotin^5^ (Supplementary Table 2). The ring synthesis reactions (Fig. 1b) were worked out many years ago largely in *E. coli* and are catalysed by four remarkably well-conserved enzymes (BioF, BioA, BioD and BioB) found throughout biology. These enzymes and BioC are annotated in several databases. However, the extant *H. pylori* genomes encode no convincing homologue of any of the known pimeloyl-ACP methyl ester esterase isozymes. An unusual feature of the *H. pylori* biotin synthetic genes is that they are scattered about the genome (Fig. 1a) rather than being organised into operons as in other bacteria. The annotations argue that, excepting BioH, *H. pylori* follows the *E. coli* pathway in which the biotin pimeloyl moiety is made by refashioning the fatty acid synthetic pathway to make pimelic acid, a dicarboxylic acid, together with the usual monocarboxylic acids^9^. The key enzyme responsible for this refashioning is the BioC methyltransferase which methylates the free carboxyl group of a fraction of the key fatty acid synthetic intermediate, malonyl-ACP^9^,^13^. This modification allows access of the intermediates to the hydrophobic active sites of the fatty acid synthetic proteins. Following completion of the pimeloyl chain the second enzyme, the BioH methyl esterase, removes the methyl group^9^,^15^. Cleavage of the methyl ester is required because the carboxyl group liberated by hydrolysis is required for the ligation of biotin to its cognate proteins^12^. *H. pylori* encodes a readily recognised BioC having 38% identity with a *Campylobacter jejuni* protein encoded within a biotin biosynthetic operon. Since these two mucosal pathogens share many properties, it seemed clear that *H. pylori* must have an esterase to remove the methyl group introduced by its cognate BioC. Given the lack of genome context of the *H. pylori* biotin genes we took a genetic complementation approach to isolate a gene encoding a protein having BioH function. This gave a pimeloyl-ACP methyl ester esterase isozyme called BioV that is found only in *Helicobacter* species and seems essential for growth of this bacterium. Due to this species specificity BioV seems a strong target for development of compounds to treat *H. pylori* infections.

## Results

### Identification of an *H. pylori* gene that functionally replaces *E. coli bioH*

**Figure 2:**
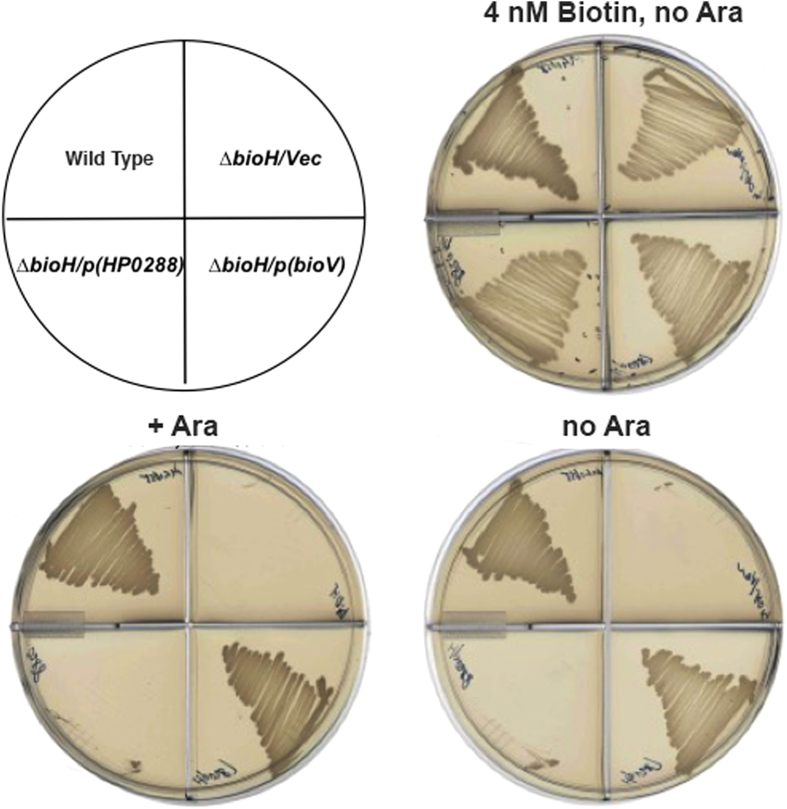
Expression of *H. pylori bioV* (HP0287) complements growth of an *E. coli* strain containing a deletion of *bioH* (Δ*bioH*). Transformants of strain STL24 (an *E. coli ∆bioH* strain) were grown at 37 °C on biotin-free medium with glycerol as carbon source. Growth in the absence of biotin was tested in either the presence or the absence of 0.02% arabinose (Ara) and comparable growth was obtained on the two media (bottom two plates). Glycerol gives basal expression of BioV whereas arabinose gives induced expression (a roughly 50-fold increase). The strains tested were: wild type (MG1655, upper left sectors), STL24 carrying plasmid pBHK565 encoding *bioV* (lower right sectors), pBHK566 encoding *HP0288* (lower left sectors) or the empty pBAD24M vector (Vec) plasmid (upper right sectors). The right hand two plates both lacked arabinose induction whereas the top plate contained biotin as a control.

Four clones were selected from the *H. pylori* genomic library that consistently complemented growth of *E. coli* STL24, a strain in which the *bioH* gene has been deleted^9^ (Supplementary Fig. 1). Upon partial sequencing all four clones were found to contain a common genomic region that contained only two open reading frames (ORFs) based on the genome sequence of *H. pylori* strain 26695. The two ORFs, HP0287 and HP0288, were annotated as a hypothetical protein and a membrane protein, respectively. To test which of these genes functioned in the *E. coli* biotin synthetic pathway, derivatives of the arabinose promoter vector pBAD24M carrying either HP0287 or HP0288 were transformed into the *∆bioH* strain STL24. The transformants were streaked onto M9 minimal media lacking biotin that contained 0.2% glycerol as sole carbon source (resulting in basal expression of the vector arabinose *araBAD* promoter), 0.05% Casamino acids and avidin (0.1 U/ml, added to bind any adventitious biotin). Plasmid-borne expression of HP0287 allowed robust growth of the *∆bioH* strain on plates that lacked biotin. Strong growth was seen in both the presence and absence of arabinose (the inducer of the *araBAD* promoter) (Fig. 2) indicating that high level expression of BioV is not toxic. In contrast expression of the HP0288 gene failed to support detectable growth of the *E. coli ∆bioH* strain (Fig. 2). Since the known BioH paralogues lack sufficient sequence similarity for alignment with the encoded protein (<10% identical residues), we have named the HP0287 gene *bioV*.

### Enzymatic activity of BioV

**Figure 3:**
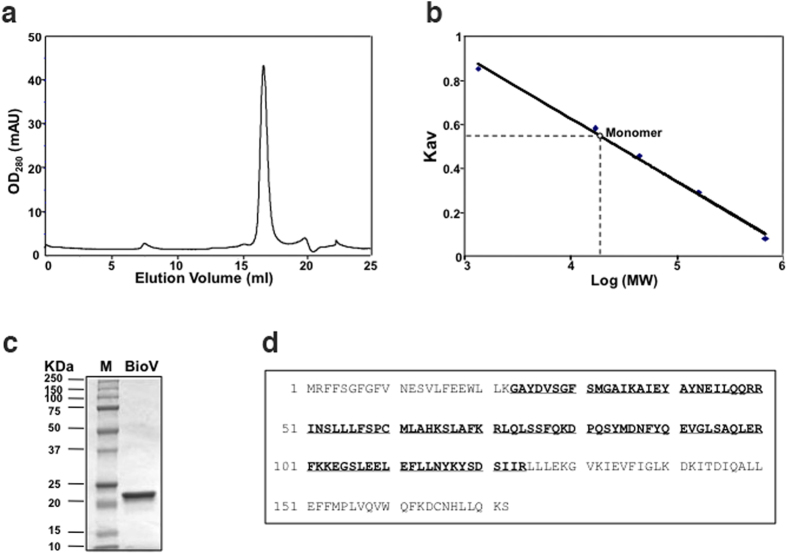
Purification and structural characterization of BioV. (**a**) Gel exclusion chromatographic profile of the hexahistidine-tagged BioV analysed on a Superdex 200HR 10/30 column (GE Healthcare) eluted at 0.4 ml min^−1^. BioV was monitored at 280 nm and eluted at 16.78 min. OD280, optical density at 280 nm; mAu, milli-absorbance units. (**b**) Determination of BioV solution structure according to elution patterns of a series of standards (Bio-Rad). The standards were vitamin B_12_ (1.35 kDa), myoglobin (horse, 17 kDa), ovalbumin (chicken, 44 kDa), γ-globulin (bovine, 158 kDa) and thyroglobulin (bovine, 670 kDa). The elution position of BioV gave an estimated molecular mass of 20 kDa based on graphic analysis of the standard curve. Kav, partition coefficient. (**c**) SDS-PAGE analysis of the purified BioV. The apparent molecular weight of His-tagged BioV is about 21 kDa. M: Molecular weight. (**d**) Mass spectrometric identification of BioV. The matching peptides are given in bold and underlined type.

To determine if BioV functions as a BioH-like esterase, we purified the protein and assayed its *in vitro* activity. An N-terminal hexahistidine tagged version of BioV was readily expressed in *E. coli* and purified to homogeneity by affinity chromatography using a nickel-chelating column followed by size exclusion chromatography. The purified protein had an apparent monomeric molecular mass of 21 kDa (Fig. 3c) making it the smallest BioH paralogue to date. The BioV size exclusion chromatographic elution profile (Fig. 3a,b) indicated that BioV is a monomer in solution as are the other three known pimeloyl-ACP methyl ester esterase isozymes. Liquid chromatography mass spectrometry analysis of tryptic peptides validated the identification of the recombinant protein with 59% coverage of the peptides predicted from the DNA sequence (Fig. 3d).

**Figure 4:**
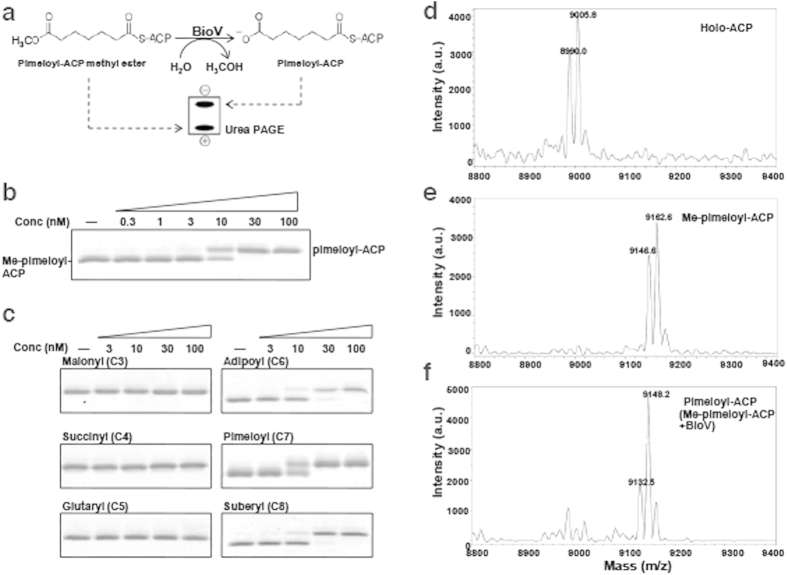
The BioV protein cleaves the ester group of pimeloyl-ACP methyl ester. (**a**) Schematic diagram of the enzymatic reaction catalysed by *H. pylori* BioV. **(b**) Enzymatic assays for *H. pylori* BioV hydrolysis of pimeloyl-ACP methyl ester to pimeloyl-ACP. The enzymatic reactions (10 μl total volume) were performed at 37 °C and contained 100 μM pimeloyl-ACP methyl ester as substrate. The product is the slower-migrating pimeloyl-ACP, which can be resolved from the substrate^9^,^17^ in a destabilizing urea-PAGE system (Materials and Methods). The minus signs denote reactions containing all components except BioV. The triangle over the right-hand six lanes of the bottom panel represents BioV concentrations in an inverse dilution series (0.3, 1, 3, 10, 30 and 100 nM). (**c**) Substrate specificity of BioV. The minus sign denotes no addition of BioV whereas the plus sign denotes addition of the enzyme. The triangle represents an inverse BioV dilution series (3,10, 30 and 100 nM). (**d**) Mass spectrometry of *H. pylori* holo-ACP. Two forms of the protein are found due to oxidization of a methionine reside to the sulphoxide during purification. Form 1 (mass 8990.0) is the native form whereas form 2 (mass 9005.8) is the oxidized form. (**e**) Mass spectrometry of pimeloyl-ACP methyl ester (Me-pimeloyl-ACP). Form 1 (mass 9146.6) is the native form whereas form 2 (mass 9162.6) is the oxidized form. (**f**) Mass spectrum of the reaction products of panel B shows cleavage of pimeloyl-ACP methyl ester (Me-pimeloyl-ACP) (9,146.2 or 9162.6 amu) to pimeloyl-ACP (mass 9,132.5 or 9148.2 amu).

BioV was assayed for the ability to convert the physiological BioH substrate, pimeloyl-ACP methyl ester, to pimeloyl-ACP using a gel electrophoretic mobility shift assay. The hydrolysis of the substrate ester moiety is indicated by the production of pimeloyl-ACP which migrates more slowly than the substrate in a destabilizing urea-PAGE system^9^,^17^. The separation results because the charge of the new ω-carboxyl group plus loss of the hydrophobic methyl ester destabilizes ACP structure and causes the ACP moiety to expand^9^,^17^. Note that *H. pylori* ACP is reported to differ from the *E. coli* and *Vibro harveyi* proteins in its pH-induced conformational transition and flexibility which may affect its interaction with various enzymes^22^. Therefore, we used *H. pylori* holo-ACP (Fig. 4d and Supplementary Fig. 2) for enzymatic synthesis of pimeloyl-ACP methyl ester. Production of pimeloyl-ACP from the methyl ester was obtained with 10 nM BioV and the reaction approached completion when the concentration of BioV was increased to 30 nM (Fig. 4b). Mass spectrometry of the reaction mixtures showed conversion of pimeloyl-ACP methyl ester to a species smaller by 14 atomic mass units (Fig. 4e,f) indicating loss of a methyl group and formation of pimeloyl-ACP. Thus BioV recognises and hydrolyzes the physiological substrate of *E. coli* BioH.

BioH is known to be a somewhat promiscuous esterase^9^,^12^,^13^,^14^. To test if this is the case for BioV, we assayed esterase activity using shorter or longer analogues of pimeloyl-ACP methyl ester as substrates. Gel mobility shift assays indicated that BioV failed to cleave malonyl-ACP methyl ester (three carbon acyl moiety) or glutaryl-ACP methyl ester (five carbon acyl moiety) (Fig. 4c). These are the two dedicated intermediates of pimeloyl moiety synthesis and thus cleavage of either molecule would abort the pathway. BioV also failed to cleave an off-pathway substrate, succinyl-ACP methyl ester (four carbon acyl moiety). Thus BioV is a relatively specific enzyme consistent with a role in biotin synthesis, although it could cleave two non-physiological substrates, adipoyl-ACP methyl ester (six carbon acyl moiety) and suberyl-ACP methyl ester (eight carbon acyl moiety). Note that prior work from this laboratory showed that induced expression of two esterases that cleaved glutaryl-ACP methyl ester was toxic to growth in biotin-free media. In contrast, strong complementation was seen at the basal level of expression^17^. Therefore, the finding that high levels of BioV expression was not toxic (Fig. 2) is consistent with the observed *in vitro* specificity.

### BioV function requires a Ser-Asp-His triad

**Figure 5:**
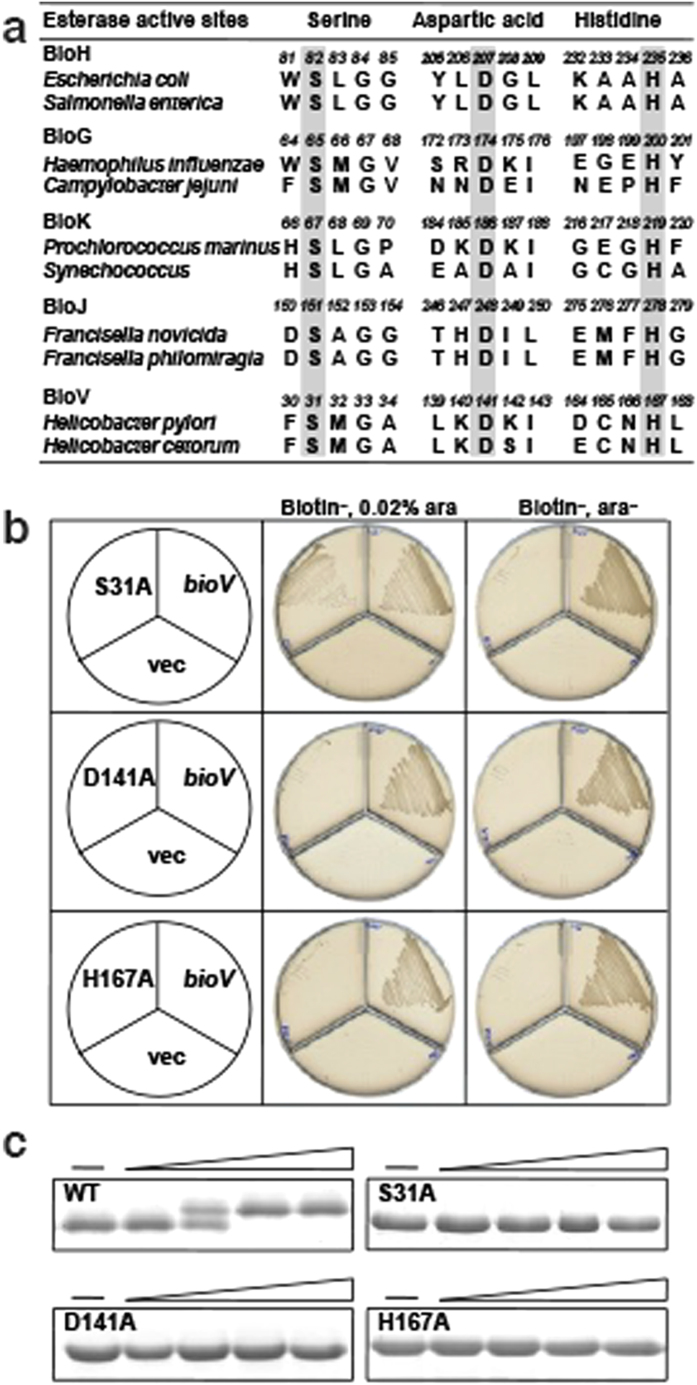
Identification of the BioV catalytic Ser-Asp-His triad residues. (**a**) Comparison of the putative active site of BioV with the catalytic residues of putative biotin synthetic esterases. The key residues are in grey. (**b**) *In vivo* functional analyses of the triad residue BioV mutant proteins. Transformants of strain STL24 (an *E. coli ∆bioH* strain) were grown at 37 °C on biotin-free medium. Growth was tested in either the presence or the absence of arabinose. The strains tested were: STL24 carrying plasmids pBHK565, pBHK624, pBHK570 or pBHK571 encoding wild type *bioV*, or, respectively, one of the mutant derivatives, S31A, D141A or H167A. The vector plasmid (vec), pBAD24M was also included. (**c**) *In vitro* functional analyses of the triad residue BioV mutant proteins. Enzymatic activities of BioV and the single mutant S31A, D141A and H167A proteins were assayed by the conformationally sensitive electrophoretic mobility shift assay. Minus denotes no addition of BioV (or a mutant protein) whereas the triangle on the right hand represents the protein levels in a inverse dilution series (3,10, 30 and 100 nM). The enzymatic reaction (10 μl total volume) contained 100 μM pimeloyl-ACP methyl ester. The reaction mixture was separated using 18% PAGE containing 2.5 M urea.

Bioinformatic analyses of the known pimeloyl-ACP methyl ester esterase isozymes (BioH, BioG, BioK and BioJ) showed that these proteins have the hallmarks of α,β-hydrolases and all contain a conserved catalytic Ser-Asp-His triad (Fig. 5a)^17^,^18^. To test the putative BioV triad residues we substituted alanine residues for the predicted residues by site-directed mutagenesis. The predicted active site residues, D141 and H167, were readily identified because substitution of these residues with alanine abolished both the *in vivo* and *in vitro* activities of the protein. The expression of both mutant enzymes failed to allow growth of the Δ*bioH* strain on biotin-free plates (Fig. 5b) and both mutant enzymes failed to cleave pimeloyl-ACP methyl ester *in vitro* (Fig. 5c). Prediction of the triad serine residue, the putative nucleophile in the cleavage reaction, was less straightforward because the N-terminal region of BioV contains several serine residues. Therefore we constructed genes encoding five singly mutant BioV proteins, S28A, S31A, S55A, S58A and S66A to test if any of these was the third active site residue. The growth of *E. coli* Δ*bioH* strains carrying a plasmid encoding each mutant protein was tested on biotin-free plates. The strain expressing the S31A mutant protein grew in the presence of the inducer, arabinose, but failed to grow in the absence of arabinose (Fig. 5b). However, the strains expressing the other four BioV mutant proteins gave wild-type growth in the presence or absence of arabinose. Thus Ser31 functioned as a catalytic residue and this was verified *in vitro* with the purified mutant protein. The S31A enzyme failed to cleave pimeloyl-ACP methyl ester (Fig. 5c) except at very high enzyme concentrations (>300 nM), a result that seems unlikely to be physiologically relevant. The residual activity seen at high BioV S31A concentrations both *in vivo* and *in vitro* could be due to one of the other serine residues present in the N-terminal region. Note that Ser31 is conserved in all BioV homologues (Supplementary Fig. 5).

### Attempted inactivation of the *H. pylori* strain 26695 *bioV* gene

We repeatedly sought to inactivate the *H. pylori bioV* gene by double crossover homologous recombination. An allelic replacement cassette consisting of the regions upstream and downstream of the *bioV* gene flanking a kanamycin resistance (*aphA*) gene from *Campylobacter coli*^23^ was constructed to give plasmids pBHK638 and pBHK639. This cassette was used to transform *H. pylori* strain 26695, but no transformants were obtained in multiple transformation experiments performed with various concentrations of biotin added to the screening media. Hence, *bioV* seemed essential for growth of *H. pylori.* As discussed below we believe this is the case because the bacterium is unable to transport exogenous biotin into the cytosol.

### Desthiobiotin synthesis requires BioV

**Figure 6:**
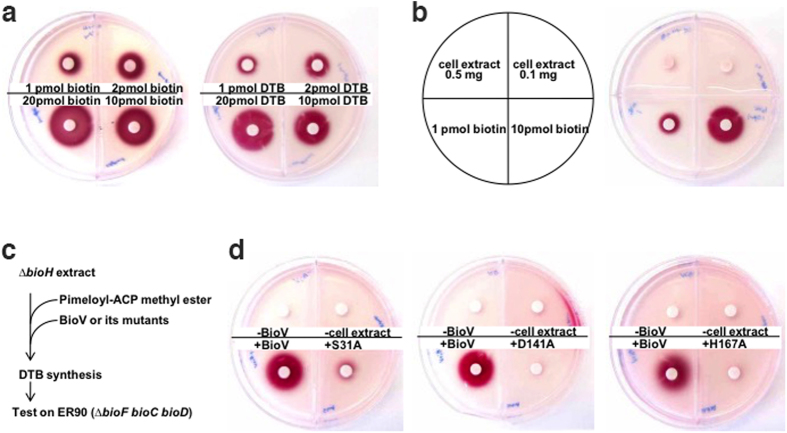
Bioassay of BioV function in the overall biotin synthesis pathway. (**a**) Very low (pmol) levels of biotin or desthiobiotin (DTB) support growth of the biotin auxotrophic strain ER90 (Δ*bioF bioC bioD*) on biotin-free minimal medium. (**b**) Evidence for the absence of biotin in cell extracts prepared from *E. coli* Δ*bioH* strain STL24 used to prepare cell extracts. The biotin indicator strain was ER90. Known amounts of biotin (positive control) or a cell extract was spotted on the paper disc as described^9^. The plates were kept at 30 °C for about 20 h. The red formazan deposit formed by reduction of the tetrazolium indicator denotes growth of the biotin auxotrophic strain ER90 which responds to either DTB or biotin. (**c**) Scheme of the *in vitro* DTB synthesis system. (**d**) Restoration of DTB synthesis to the *∆bioH* extract by addition of pimeloyl-ACP methyl ester and BioV. Either the purified wild type BioV or one of the mutant BioV derivatives (S31A, D141A and H167A) was added to 20 μg ml^−1^ (0.5 μM). The upper left quadrant reactions contained all components required for DTB synthesis except BioV (or a mutant derivative) whereas the samples spotted on the upper right quadrants lacked cell extract and contained only both the wild type and mutant BioV proteins. The reactions of the lower quadrants contained one of the BioV proteins and all components required for DTB synthesis. The plus sign denotes addition of BioV (or a mutant derivative); whereas the minus sign denotes no addition of BioV (or a mutant derivative) or no cell extract.

To further test the role of BioV in biotin synthesis we used the desthiobiotin (DTB) biosynthesis assay. (Note that the penultimate intermediate, DTB, was assayed rather than biotin in order to avoid the complication of the extremely unstable BioB enzyme responsible for the last step of the pathway^24^,^25^). The previously reported *in vitro* cell-free extract system^9^ that converts malonyl-CoA to DTB was modified to a coupled system in which crude extract of a Δ*bioH* strain was assayed. In the bioassay, which can reliably detect 1 pmol of DTB or biotin (Fig. 6a), the test solution diffuses from a filter disk into biotin-free minimal-medium agar seeded with the strain ER90 (Δ*bioF bioC bioD*), an *E. coli* strain that requires either DTB or biotin for growth. If growth occurs, a redox indicator becomes reduced and forms a bright red, insoluble deposit, the area of which is proportional to the concentration of the biotin pathway product. Addition of pimeloyl-ACP methyl ester to crude extracts prepared from the Δ*bioH* mutant strain (0.1–0.5 mg total protein) gave no detectable growth of the assay strain (Fig. 6b) whereas a good growth response was seen upon assay of reactions containing purified BioV. Hence, BioV addition gave DTB synthesis (Fig. 6d). In contrast, no DTB synthesis was detected when the incubations contained any of the mutant BioV proteins (Fig. 6d). These results clearly demonstrated that BioV can function in bacterial DTB biosynthesis.

### *Helicobacter* species encode a novel carboxylesterase paralogue

*H. pylori* is the primary *Helicobacter* species studied because of its importance in human medicine. However, non-*pylori Helicobacter* species (NPHS), which naturally inhabit mammals and birds, have been reported and detected in human clinical specimens^26^,^27^. Protein sequence alignments showed that genes encoding close homologues to BioV are found in six other *Helicobacter* species (Supplementary Fig. 5). We tested two of these homologues by cloning the candidate genes of *H. acinonychis* (Hac_0547) and *H. cetorum* (HCD_02280) into vector pBAD24M. Expression of both genes allowed growth of the *E. coli* Δ*bioH* strain on biotin-free minimal medium (Supplementary Fig. 4).

**Figure 7:**
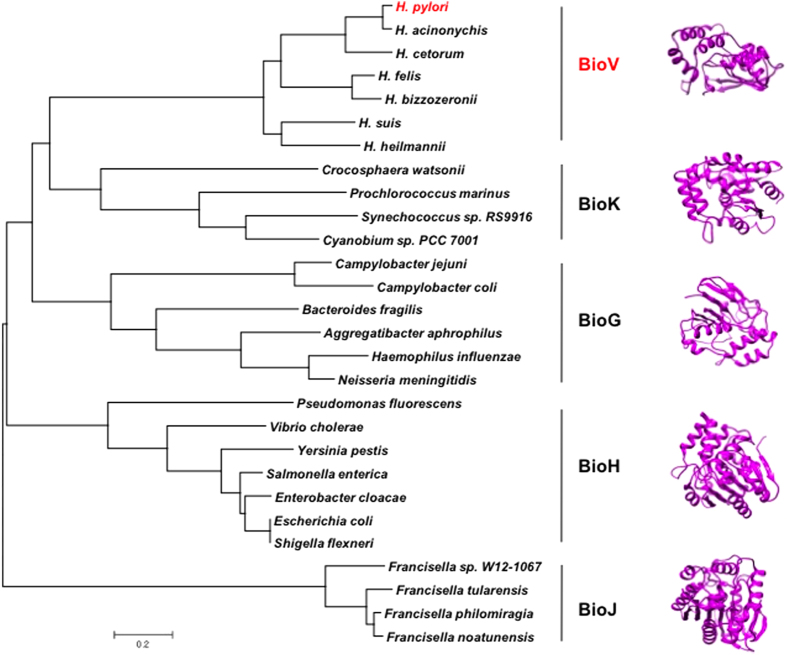
Phylogeny and structural modelling of the bacterial esterases that cleave pimeloyl-ACP methyl ester. Phylogenetic analyses were conducted by the minimum-evolution method using MEGA6. On the right side of the figure the modelled structure of each esterase is shown together with the known structure of BioH^14^,^15^. *H. pylori* BioV is coloured red.

Although the BioV proteins are predicted to be members of the α,β-hydrolase family^28^,^29^, they seem evolutionarily distinct from the four known pimeloyl-ACP methyl ester cleavage enzymes (BioH, BioG, BioK and BioJ). BioV is thirty residues shorter than the shortest of the known proteins (BioG) and shares essentially no sequence identity with the other proteins. Given the fact that these isozymes recognise the same physiological substrate (pimeloyl-ACP methyl ester) and share the same catalytic residues^17^,^18^, we constructed a minimum-likelihood phylogenetic tree and found that these proteins were placed into five different subclades. The BioV homologues are restricted to *Helicobacter* species and form a subclade distinct from other groups (Fig. 7). Moreover, based on protein structure predictions from threading algorithms, all five groups of proteins appear structurally distinct (Fig. 7) consistent with the diversity in their phylogeny. Note that, although all but BioH are only modelled structures, the threading predictions are likely to be reasonably accurate because they are based on an unusually large data set (the Protein Data Base contains over 1500 structures of α,β-hydrolase family esterases). We posit that BioV represents a new class of biotin synthesis carboxylesterase.

## Discussion

The enzymes responsible for synthesis of the biotin heterocyclic rings are strongly conserved throughout biology whereas the enzymes that catalyse the last step in pimeloyl moiety synthesis show remarkable diversity. The enzymes that catalyse cleavage of pimeloyl-ACP methyl ester seem to be the “wild cards” of biotin synthesis in that five different enzymes (BioH, BioG, BioK, BioJ and BioV) have been shown to catalyse this key physiological reaction^16^,^17^. To date all are members of the α,β-hydrolase family, an enormous protein family that has been subdivided into many subfamilies, clans and clades. The Pfam database places these enzymes into different families within the same clan (CL0028). BioV, like BioH and BioG, is an α,β-hydrolase family 6 protein whereas BioK and BioJ are in α,β-hydrolase families 5 and 3, respectively. The core of each enzyme is an α,β-sheet that contains eight β-strands connected by six α-helices. These enzymes each have a catalytic triad with the triad residues located on loops.

Two of the pimeloyl-ACP methyl esterases, BioH and BioG, are widely distributed among diverse bacteria whereas the three remaining enzymes currently appear restricted to specific species of bacteria^16^,^17^. BioV is found only in *Helicobacter* species, BioJ only in *Francisella* species^30^ and BioK only in cyanobacteria^16^. In most of these bacteria BioC, the partner of the pimeloyl-ACP methyl ester esterase, is in a putative operon containing most, if not all, of the enzymes required for biotin heterocyclic ring synthesis. A *bioH* gene is sometimes found within the operon^16^,^17^. If so, it is always the gene immediately upstream of *bioC*. However in other cases such as *bioV* in *H. pylori* and *bioH* in *E. coli*, the hydrolase is encoded at a genome location far removed from the other biotin synthetic genes. One possibility for this disconnect between the operon location of *bioC* and the distantly located hydrolase genes is that methylation of malonyl-ACP must be tightly controlled because excess methylation blocks fatty acid synthesis^13^. In contrast, as long as the hydrolase is reasonably specific for pimeloyl-ACP methyl ester, high-level production may be benign. Indeed, the BirA repressor that tightly controls transcription of the other biotin synthetic genes does not regulate *E. coli bioH*^31^,^32^. Another consideration is that methyl transfer requires binding and stabilization of S-adenosyl-L-methionine, an extremely reactive substrate, followed by delivery of the methyl group to a second substrate. In contrast ester cleavage is a much simpler reaction since only a single substrate (plus water) is involved. In this scenario BioC is a “real” biotin enzyme whereas the hydrolases appear to have been recruited in a rather *ad hoc* manner after acquisition of *bioC* and the genes that encode the biotin heterocyclic ring synthesis enzymes. One possibility is that an esterase involved in another pathway gained the ability to cleave pimeloyl-ACP methyl ester while retaining its original activity, a process that is called “moonlighting”^33^,^34^. Gene duplication and further mutagenesis can result in two specific enzymes, one catalysing the original activity and second specific for pimeloyl-ACP methyl ester cleavage^33^,^34^. This scenario is facilitated by the fact that only very modest esterase activity would be needed since biotin is required only in tiny amounts (*E. coli* growth requires only a few hundred biotin molecules/cell^35^). As noted above, in some bacteria the hydrolase gene has been incorporated into the biotin synthesis operon. An extreme case is *Bacteriodies fragilis* which encodes a BioG-BioC fusion protein that functions in both reactions^17^. Within these biotin operons the coding sequence of hydrolase gene often overlaps that of *bioC* and thus the translation as well as the transcription of these genes appears “hard-wired”. The *H. pylori* genome is atypical in that its biotin synthetic genes are scattered about the genome. This is in marked contrast to *C. jejuni* where *bioG* and all of the other biotin synthetic genes except *bioB* are found in a putative operon. Indeed, the pattern of scattered *bio* genes and the presence of *bioV* are not found in all bacteria currently placed in the genus Helicobacter. Both *H. cinaedi* and *H. hepaticus* have putative *bio* operons that contain a *bioG* gene and resemble that of *C. jejuni.* This is particularly true of *H. hepaticus* where the *bioG* gene is located immediately upstream of the *bioC* gene. These findings may speak to the unsettled nature of Campylobacteracae taxonomy over the past decades.

The fact that each of the four *bioH* complementing clones isolated had different endpoints argues that the clone bank we screened was highly random and contained several genome equivalents. Thus, BioV seems likely to be the sole *H.* pylori enzyme able to cleave pimeloyl-ACP methyl ester. Our inability to delete *bioV* from the genome of *H. pylori* strain 26695 is consistent with the results of others. Salama and coworkers^36^,^37^ have mapped over 5300 transposon insertions into several *H. pylori* genomes without detection of an insertion into HP0287 (*bioV*), although the growth media contained rich sources of biotin and insertions into the flanking genes were isolated (N. Salama, personal communication). Genomic data also argue that HP0287 (*bioV*) is essential. This gene is strictly conserved in the genomes of *H. pylori* strains isolated from extremely diverse geographical, ethnic and clinical origins (we collated literature microarray and genome sequencing data for over 175 strains)^38^,^39^,^40^,^41^. Extrapolation of the numbers of core (presumably essential) genes among these strains ranges from 786 to about 1100 illustrating the unusual plasticity of *H. pylori* genomes and the high rate of flux of genes in and out of these genomes. Moreover, in experimental infections of human volunteers *bioV* was retained in all 30 patients for the duration of the study^42^. Given the remarkably high rates of mutation and recombination in this bacterium, it seems likely that if HP0287 (*bioV*) were nonessential for growth, it would have been lost from at least some of these strains.

We believe the essential nature of *bioV* is due to the inability of *H. pylori* to transport biotin into the cytosol. It seems likely that *H. pylori* has not developed or acquired a biotin transporter because its harsh gastric habitat does not contain free biotin. Mammals lack the ability to make biotin and must obtain it from the diet. However, dietary biotin is not found in the free form but rather as biotin covalently attached to its cognate proteins. Free biotin is generated by biotinidase, an enzyme that cleaves the biotin-protein amide linkage^43^. At the low pH of the gastric environment biotinidase would be inactivated. Moreover, the rapid peristaltic transit of food through the stomach would impede chemical cleavage of the biotin-protein amide bond, a linkage that is highly stable to acidic conditions. Consistent with the hypothesis that free biotin is not available in its habitat, *H. pylori* lacks recognisable homologues of the known proteobacterial biotin transporters. Another indication that *H. pylori* lacks access to environmental biotin is that it seems unable to regulate biotin synthesis in response to environmental biotin. In *E. coli* the regulation is dictated by BirA, an unusual protein that it is both the repressor of *bio* operon transcription and a biotin protein ligase (the enzyme that attaches biotin to its cognate enzyme subunits)^44^,^45^,^46^. When an excess of biotin is present, the biotinoyl-5′-AMP-liganded form of BirA protein occupies the *bio* operator and effectively represses transcription of the biotin operon genes^5^,^47^. Operator DNA binding requires an N-terminal winged helix-turn-helix domain which the *H. pylori* biotin protein ligase (HP1140) is lacking. Therefore *H. pylori* should be unable to regulate biotin synthesis in response to environmental biotin^45^,^48^.

Given the worldwide explosion of antibiotic resistance, targeting novel aspects of central metabolism in bacterial pathogens represents an attractive proposition in the future development of novel antibacterial drugs^49^. Folate biosynthesis was the first established anti-infective target for antituberculosis drugs^50^,^51^. Biotin biosynthesis has subsequently been shown to be required for the establishment and maintenance of infections by *M. tuberculosis*^7^,^52^ and *F. tularensis*^8^. Colonization and infection by enterohemorrhagic *E. coli* also depend on intestinal biotin levels^53^. Thus understanding the mechanism of biotin is synthesis in *H. pylori* could provide targets for the development of antimicrobials to combat infection, particularly since the bacterium seems unable to access the host biotin supply. BioV is unique to *Helicobacter* species and thus BioV inhibitors should not otherwise affect the host microbiome. In contrast, targeting the other biotin synthetic enzymes could damage the microbiome because those proteins are highly conserved.

## Materials and Methods

### Bacterial strains, plasmids and growth conditions

Bacterial strains, plasmids, and oligonucleotides used are given in Supplementary Table 1. *E. coli* was grown at 37 °C in Luria–Bertani (LB) medium (tryptone, 10 g l^−1^; yeast extract, 5 g l^−1^; NaCl, 10 g l^−1^; pH 7.0). The biotin-free M9 minimal media containing 0.05% vitamin-free Casamino Acids plus avidin (0.1 U/ml) and 0.2% glycerol as sole carbon source was used to test for biotin requirements. Required antibiotics were added (in μg ml^−1^): sodium ampicillin, 100; kanamycin sulfate, 50; gentamicin sulfate, 10 and chloramphenicol, 20. L-arabinose was added at a final concentration of 0.02%. Bacterial growth was determined by optical density at 600 nm. Oligonucleotide primers were synthesized by Integrated DNA Technologies and cloned genes were verified by sequencing performed by ACGT, Inc. Gene sequences were amplified using Pfu Turbo DNA polymerase (Stratagene) according to the manufacturer’s recommendations. *H. pylori* strain 26695 genomic DNAs from the American Type Culture Collection was used as templates. Primers bioV-f and bioV-r were used to amplify *bioV* (HP0287), and HP0288-f and HP0288-r for HP0288. The PCR products were inserted into plasmid pBAD24M cut with NdeI and SalI using T4 DNA ligase (New England Biolabs) to generate the plasmids, pBHK565 and pBHK566. The plasmids pZL394 carrying *HCD_02280* (*HcbioV*) and pZL395 *Hac_0547* (*HabioV*) were constructed using the same method with primer sets HbioV-f/HcbioV-r and HbioV-f/HabioV-r, respectively. Plasmid pBHK531 expressing *H. pylori* apo-ACP was obtained by use of primers HPAcpP-f and HPAcpP-r for insertion into pET28b. Plasmid pBHK532, a pBAD33-derivative that expresses HPAcpS, was obtained by use of primers HPAcpS-f and HPAcpS-r. Qiagen provided plasmid isolation and PCR product purification kits.

### Plasmid library construction and screening for complementation of an *E. coli ∆bioH* strain

Genomic DNA from cells of the *H. pylori* strain 26695 was extracted using the Wizard Genomic DNA Purification Kit (Promega). A plasmid library was constructed by ligation of genomic DNA, partially digested by Sau3AI, into the BamHI site of pTRKL2^54^,^55^, which was dephosphorylated by calf-intestinal alkaline phosphatase (New England Biolabs). The size of cloned inserts ranged from 4 to 10 kb. The ligation products were then transformed into *E. coli* DH5α with selection on BHI plates containing X-gal and 150 μg/ml erythromycin. Isolated white *E. coli* colonies (about 10,000) were collected and the mixture of recombinant plasmids was purified using the Qiagen Spin Miniprep kit. This number of colonies should cover the *H. pylori* genome several fold assuming a random distribution of Sau3AI sites. The extracted plasmids were electroporated to the *E. coli* Δ*bioH* strain STL24. The 8,000 clones obtained were screened for growth on biotin-free M9 medium containing 0.05% Casamino Acids and 0.2% glycerol plus avidin (0.1 U/ml). The plasmids isolated from the growth-complemented strains were again transformed into strain STL24 to confirm complementation. Finally the plasmids confirmed to complement were sequenced and the genomic locations of the inserts identified by BLAST searches against the *H. pylori* 26695 genome.

### Protein expression and purification

The *bioV* gene amplified from *H. pylori* strain 26695 genomic DNA was inserted into vector pET-28b to give plasmid pBHK555 which encodes BioV with a N-terminal hexahistidine (His)-tag. BioV was expressed in Rosetta (DE3) pLysS grown at 37 °C in LB medium. At an OD_600_ of 0.8, the cultures were induced with 0.15 mM isopropyl-β-D-thio-D-galactoside (IPTG) and grown at 30 °C for an additional 5 h prior to harvest. The cells were collected, resuspended in lysis buffer (50 mM sodium phosphate, 300 mM NaCl, 10 mM imidazole, 1 mM dithiothreitol, pH 8.0), lysed by French pressure cell treatment and centrifuged. The clarified bacterial supernatant was loaded onto a nickel-ion affinity column (Qiagen). The column was washed with a 50 mM NaH_2_PO_4_ (pH 8.0) buffer containing 300 mM NaCl, 40 mM imidazole and 1 mM dithiothreitol, to remove contaminant proteins. The His-tagged BioV protein was eluted in the same buffer containing 200 mM imidazole. The protein was concentrated by ultrafiltration (10 kDa cutoff) and exchanged into a sodium phosphate buffer (50 mM NaH_2_PO_4_, 150 mM NaCl, 1 mM dithiothreitol, pH 8.0). The protein purity was visualized by gradient SDS-PAGE (4–20%), and further confirmed by liquid chromatography quadruple time-of-flight (qTOF) mass spectrometry of tryptic peptides as described previously^54^. The solution structure of BioV was analysed by size exclusion chromatography on a Superdex 200 10/300 GL column (GE Healthcare) using an AKTA Purifier10 at 0.4 ml/min in phosphate running buffer (135 mM NaCl, 2.7 mM KCl, 1.5 mM Na_2_HPO_4_, and 8 mM K_2_HPO_4_, 10% glycerol, pH 7.6). The *H. pylori* apo-ACP was expressed in the *E. coli* Rosetta (DE3) pLysS strain transformed with pBHK531 carrying the *H. pylori acpP* gene and the holo-ACP was expressed in the same host strain using the two plasmids, pBHK531 and pBHK532 carrying the *H. pylori acpS* gene. The *H. pylori* apo-ACP and holo-ACP were purified as described previously^56^.

### Site-directed mutagenesis of *bioV*

Plasmids pBHK624, pBHK570 and pBHK571 each carrying a single mutation within the BioV coding sequence were obtained using the QuickChange mutagenesis kit with pBHK565 as the PCR template. The primers used in PCR and mutagenesis are listed in Supplementary Table 1. The constructed plasmids were transformed into *E. coli* DH5α by CaCl_2_ treatment. The mutations were verified by DNA sequencing. These three BioV mutant plasmids were then introduced into *E. coli* Δ*bioH* strain STL24 for test for complementation ability. Plasmids pBHK616, pBHK576 and pBHK577 each carrying a single mutation were obtained using the QuickChange mutagenesis kit with pBHK555 as the PCR template.

### Esterase activity assays

The reactions contained 100 mM sodium phosphate (pH 7.6), 100 μM pimeloyl-ACP methyl ester, and different concentrations of BioV or its mutant derivatives. A premix of buffer and the ACP substrate, lacking BioV, was incubated at 37 °C for 2 min. The hydrolysis reaction was initiated by adding BioV or (its mutant derivatives) and incubated at 37 °C for 10 min. Then the reaction was sampled, immediately quenched by addition of an equal volume of 10 M urea, and stored on dry ice. The reaction samples were loaded into 18% PAGE gel containing 2.5 M urea and then run at 130V for 2.5 h. The monomethyl esters of the various dicarboxylic acids were synthesized and converted to ACP thioesters using *Vibro harveyi* acyl-ACP synthetase AasS^57^ and *H. pylori* holo-ACP as described previously^15^.

### Protein mass spectrometry

The solutions of acyl-ACP derivatives from the reconstituted assays described above were loaded in binding buffer (25 mM 4-morpholineethanesulfonic acid, pH 6.1) containing 100 mM LiCl and the column was washed twice with the same buffer containing 250 mM LiCl. Acyl-ACPs were eluted in binding buffer containing 500 mM LiCl followed by dialysis using a 3,500 molecular weight cut-off membrane against 2 mM ammonium acetate at 4 °C for 15 h. The extracts were dried under a stream of nitrogen and 2 μg samples were dissolved in 20 μl 50% acetonitrile containing 0.1% formic acid. Mass spectral analyses were performed by the University of Illinois Mass Spectrometry Laboratory. The mass spectra were collected in positive ion mode on an UltrafleXtreme MALDI TOF/TOF mass spectrometer (Bruker Daltonics) equipped with a frequency tripled Nd–YAG solid state laser using the FlexControl 1.4 software package (Bruker Daltonics). Following external calibration, 2000 spectra were acquired at 500 Hz using a randomized raster, summed, and saved for analysis. Data processing was done using the FlexAnalysis 3.4 software package (Bruker Daltonics). Spectra were smoothed and a baseline correction was applied using the software package features.

### Preparation of Δ*bioH* cell-free extracts

Strain STL24 (*E. coli* MG1655 Δ*bioH*) was grown at 37 °C to 0.8 OD at 600 nm in 250 ml of minimal medium containing 2 nM biotin. The cells were washed in M9 salts medium to remove biotin and subcultured into 1 liter of glucose minimal medium at 37 °C for 5 h to derepress *bio* operon transcription by starvation for biotin. The cells were lysed in assay buffer by French Press treatment at 17,500 p.s.i. followed by centrifugation at 20,000 × *g* for 20 min to obtain the soluble fraction of the cell extract. Ammonium sulfate was added slowly to 85% of saturation to the soluble cell extract in a beaker under constant stirring on ice until completely dissolved. This step was intended to remove small molecules and deplete the extracts of ACP, which remains soluble in such ammonium sulfate solutions. The protein precipitant was collected by centrifugation at 10,000 × *g* and stored at −80 °C. The precipitant was solubilized before use by dialysis in 7,000 molecular weight cut-off membranes against assay buffer at 4 °C for 3 h to remove ammonium sulfate and any remaining small molecules.

### *In vitro* DTB synthesis

An *in vitro* system that utilized crude extracts of strain STL24 (*E. coli* Δ*bioH*) was reconstituted to test the potential role of BioV in biotin biosynthesis, as described by Lin *et al.*^9^ with minor changes. The production of DTB was visualized using the biotin auxotrophic strain ER90 (Δ*bioF bioC bioD*)-based biotin bioassay previously developed^9^. This assay allows *in vitro* conversion of ACP-bound substrate into DTB using enzymes in cell-free extracts. A 100 μl reaction in assay buffer contained 1 mg of *ΔbioH* cell-free extract protein, 1 μmol MgCl_2_, 0.5 μmol dithiothreitol, 0.01 μmol pyridoxal-5′-phosphate, 50 μg pimeloyl-ACP methyl ester, 0.1 μmol L-alanine, 0.1 μmol KHCO_3_, 0.1 μmol NADPH, 0.1 μmol ATP, 0.1 μmol glucose-6-phosphate and 0.1 μmol SAM. The reactions were incubated at 37 °C for 3 h and quenched by immersion in boiling water for 10 min. DTB production was bioassayed as follows. *E. coli* strain ER90 (Δ*bioF bioC bioD*) was grown in 5 ml of glucose M9 minimal medium containing 2 nM biotin at 30 °C overnight. The cells were washed with M9 medium and subcultured in 100 ml of glucose minimal medium at 37 °C for 5 h to starve the cells for biotin. The cells were collected by centrifugation, washed again in M9 medium and mixed into 150 ml of glucose minimal agar containing the redox indicator 2,3,5-triphenyl tetrazolium chloride (0.1%, w/v) to a final OD at 600 nm of approximately 0.1. Six ml of the mixture was poured into Petri dishes sectored with plastic walls to prevent cross-feeding. A 6 mm paper disk (BBL) was placed upon the agar, and the disk was spotted with 10 μl of a reaction to be tested. After incubation of the plates at 30 °C overnight, growth of strain ER90 was visualized as a red deposit of formazan.

### Bioinformatic analyses

The multiple alignments of BioV proteins were conducted using the ClustalW2 program (http://www.ebi.ac.uk/Tools/clustalw2/index.html), and final output was processed by the ESPript 2.2 server (http://espript.ibcp.fr/ESPript/cgi-bin /ESPript.cgi). Minimum evolution phylogenetic trees were inferred with Mega6 program^58^. The statistical robustness and reliability of the branching order within each phylogenetic tree were confirmed with a bootstrap analysis using 1000 replicates. Sequences to be analysed were retrieved from the NCBI microbial protein database http://www.ncbi.nlm.nih.gov/sutils/genom_table.cgi). The amino acid sequence of *H. pylori* BioV and other pimeloyl-ACP methyl ester cleavage enzymes were submitted to the CPHmodels 3.0 Server (http://www.cbs.dtu.dk/services/CPHmodels), generating a PDB file of the modelled structure, which searches for a reasonable template of known structure. The tertiary structure models of pimeloyl-ACP methyl ester esterases were obtained using Swiss PDBViewer 4.0.1 software from the Swiss Institute of Bioinformatics (http://spdbv.vital-it.ch/).

## Additional Information

**How to cite this article**: Bi, H. *et al.* A Biotin Biosynthesis Gene Restricted to *Helicobacter. Sci. Rep.* **6**, 21162; doi: 10.1038/srep21162 (2016).

## Supplementary Material

Supplementary Information

## Figures and Tables

**Figure 1:**
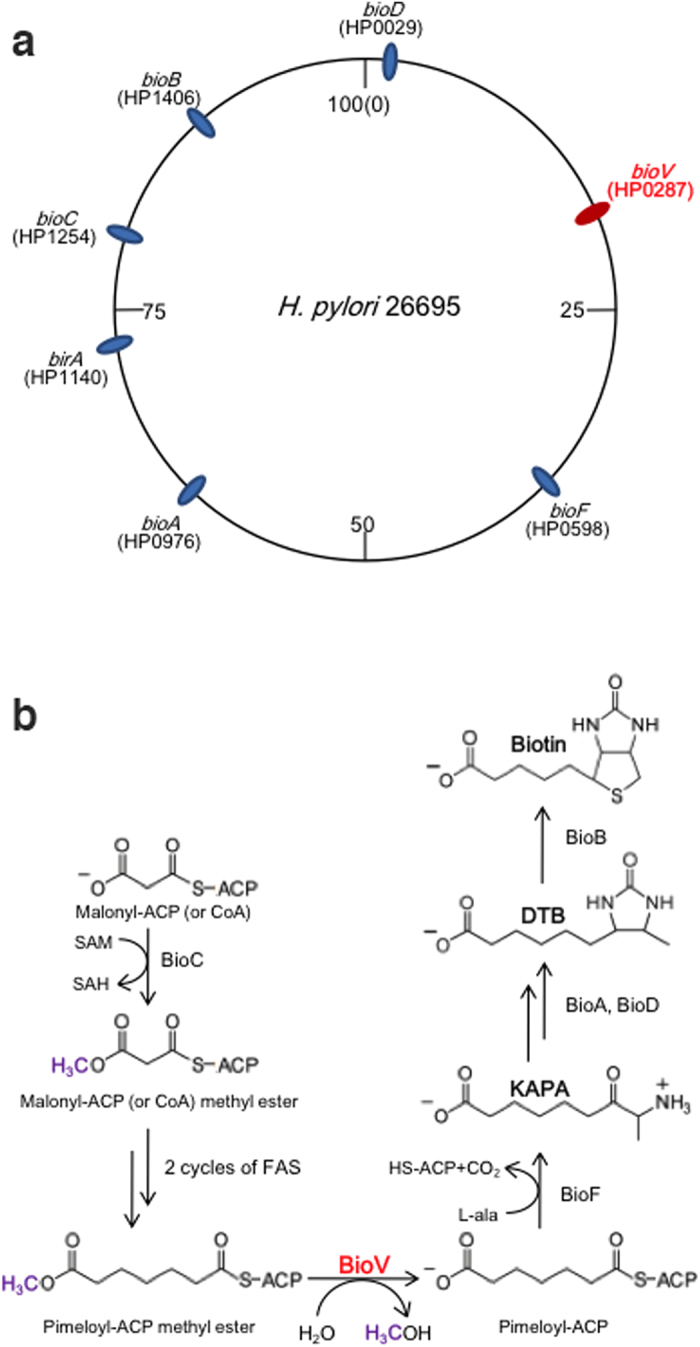
Genetic organisation of biotin synthesis and related genes and the proposed model for the *H. pylori* biotin biosynthesis pathway. (**a)** The genomic locations of the biotin biosynthesis genes are shown. The *bioV* gene is coloured red and the other genes are coloured black. (**b**) Scheme of the proposed *H. pylori* biotin synthesis pathway. FAS denotes the fatty acid synthesis cycle. Pimeloyl-ACP methyl ester can also be called methyl pimeloyl-ACP.
