# Supplementary material for: A Biotin Biosynthesis Gene Restricted to Helicobacter
Source: Sci Rep. 2016 Feb 12;6:21162. doi: 10.1038/srep21162 (PMC4751477; doi:10.1038/srep21162)
Supplement: Supplementary Information [file srep21162-s1.pdf]

Supplement to:

A Biotin Biosynthesis Gene Restricted to *Helicobacter*

Hongkai Bi[1,2]*, Lei Zhu[3], Jia Jia[1,2] and John E. Cronan[3,4]*

[1]Department of Pathogenic Biology, [2]Key Laboratory of Pathogen Biology of Jiangsu Province, Nanjing Medical University, 140 Hanzhong Road, Nanjing, Jiangsu 210029, China
Departments of [3]Microbiology and [4]Biochemistry, University of Illinois, B103 Chemical and Life Sciences Laboratory, 601 S. Goodwin Ave, Urbana, Illinois 61801, USA.

Supplementary Table 1. Bacterial strains and plasmids used in this study

Bacterial Strains	Genotype	Reference or source
E. coli		
DH5α	Δ(*argF–lac*)169 φ80d*lacZ58*(M15) *ΔphoA8 glnV44 deoR481 gyrA96, recA1 endA1, hsdR17*	Lab stock
BL21 (Tuner)	F^- *ompT hsdS*$_B$ (r_B^- m_B^-) *gal dcm lacY1*	Novagen
Rosetta(DE3) pLysS	F^- *ompT hsdS*$_B$ (r_B^- m_B^-) *gal dcm* (DE3) pLysS	Novagen
STL24	MG1655, Δ*bioH*	[1]
ER90	Δ*bioF bioC bioD*	[2,3]
H. pylori 26695	Wild-type strain	[4]
**Plasmids**		
pET-28b	Km^r, T7 promoter-based expression vector, pBR322 origin	Novagen
pBluescript SKII	Amp^r, cloning vector	Stratagene
pHel2	Cm^r, *E. coli-H. pylori* shuttle vector	[5]
pTRKL2	Erm^r, low-copy-number *E. coli/Lactococcus* shuttle vector	[6]
pBAD24M	Amp^r, NcoI site of pBAD24 changed to an NdeI site, modified pBR322 origin	[7]
pBAD33	Cm^r, expression vector containing P_{BAD} promoter and *araC*, pACYC184 origin	[8]
pBHK565	Amp^r, *HP0287* (*bioV*) cloned into the NdeI and SalI sites of pBAD24M	This work
pBHK566	Amp^r, *HP0288* cloned into the NdeI and SalI sites of pBAD24M	This work
pBHK555	Km^r, *bioV* cloned into the NdeI and SalI sites of pET-28b	This work
pBHK624	Amp^r, BioV S31A of BHK565	This work

pBHK570	Amp^r, BioV D141A of BHK565	This work
pBHK571	Amp^r, BioV H167A of BHK565	This work
pBHK616	Km^r, BioV S31A of BHK555	This work
pBHK576	Km^r, BioV D141A of BHK555	This work
pBHK577	Km^r, BioV H167A of BHK555	This work
pBHK532	Cm^r, PCR-amplified *HpAcpS* inserted between the KpnI and HindIII sites of pBAD33	This work
pBHK531	Km^r, PCR-amplified *HpAcpP* inserted between the NcoI and HindIII sites of pET28-b	This work
pBHK638	Amp^r, Km^r, pBluecript SKII (+) containing a cassette consisting of the regions upstream and downstream 600 bp of the *bioV* gene flanking the kanamycin resistance (*aphA3*) gene	This work
pBHK639	Cm^r, Km^r, pHel2 containing the cassette consisting of the regions upstream and downstream 600 bp of the *bioV* gene flanking the kanamycin resistance (*aphA3*) gene	This work
pZL394	Amp^r, *HCD_02280* (*HcbioV*) cloned into the NdeI and SalI sites of pBAD24M	This work
pZL395	Amp^r, *Hac_0547* (*HabioV*) cloned into the NdeI and SalI sites of pBAD24M	This work
**Oligonucleotides**	**Sequence**	
bioV-f	ATCTGCCATATGCGTTTTTTAGTGGTTTTGG	
bioV-r	ACTCTGTCGACTTAAGATTTTGCAACAAATGG	
HP0288-f	CGTCGGCATATGAAAAAATTTGGTTTGG	
HP0288-r	ATTCTGTCGACTTAAAGCACATTTTTTCCAAGC	
HbioV-f	CAGGTCCATATGCGTTTTTTAGTGGTTTTG	

HabioV-r	ATCGTGTCGACTTAAGATTTTTGCAACAAATGG	
HcbioV-r	ATCTTGTCGACTTATTTCCACCCTTTTGATTC	
HPAcpS-f	AATTAGAGCTCAGGAGTAAACGAATGATTGGCATAGATATTG	
HPAcpS-r	GCGCGGGTACCTTATTCATTTGACGAAGAAAC	
HPAcpP-f	ACTGAACCATGGGCATGGCTTTATTTGAAGATATTC	
HPAcpP-r	GGCCAAGCTTAAGCCAGTTTATTATCCTC	
S31A-f	CCTTAATCGCACCCATAGCAAAGCCTGACACATCATA	
S31A-r	TATGATGTGTCAGGCTTTGCTATGGGTGCGATTAAGG	
D141A-f	AAAAGGGCTTGAATGTCAGTGATTTTAGCTTTTAAACCGATAAACACTTCAATC	
D141A-f	GATTGAAGTGTTTATCGGTTTAAAAGCTAAAATCACTGACATTCAAGCCCTTTT	
H167A-f	CGACTTAAGATTTTTGCAACAAAGCGTTGCAATCCTTAAACTGCCACA	
H167A-r	TGTGGCAGTTTAAGGATTGCAACGCTTTGTTGCAAAAATCTTAAGTCG	
BioVmut1	AATGACTCGAGAATGAAAGCGCTCTAAACG	
BioVmut2	ATAGAGTCGACTTCACCCGTTCAATAAATCAT	
BioVmut3	ATAAAGTCGACGCGAACCATTTGAGGTGATAG	
BioVmut4	TTTCATCTTCCACCTTTTTTAAAACAATTCATCCAGT	
BioVmut5	ACTGGATGAATTGTTTTAAAAAAGGTGGAAGATGAAA	
BioVmut6	ATTATGGATCCTGCTACCCTCTCATTTCCTA	

The underlined sequences are restriction sites.

Supplementary Table 2. *H. pylori* biotin and fatty acid synthesis genes

**Genes/genetic loci**	**Enzyme activity**
**Biotin biosynthesis and attachment**	
bplA (HP1140)	Biotin protein ligase
bioC (HP1254)	SAM-dependent malonyl-ACP *O*-methyltransferase
bioV (HP0287)	Pimeloyl-ACP methyl ester esterase
bioF (HP0598)	8-amino-7-oxononanoate synthase
bioA (HP0976)	7,8-diaminononanoate synthase
bioD (HP0029)	Desthiobiotin synthase
bioB (HP1406)	Biotin synthase, SAM radical enzyme
**Fatty acid biosynthesis**	
accA (HP0557)	Carboxyl-transferase subunit
accB (HP0371)	Biotin carboxyl carrier protein (BCCP)
accC (HP0370)	Biotin carboxylase
accD (HP0950)	Carboxyl-transferase subunit
acpP (HP0559)	apo-ACP
acpS (HP0808)	Holo-ACP synthase
fabD (HP0090)	Malonyl-CoA : ACP transacylase
fabF (HP0558)	3-Ketoacyl-ACP synthase II
fabH (HP0202)	3-Ketoacyl-ACP synthase III
fabG (HP0561)	3-Ketoacyl-ACP reductase
fabZ (HP1376)	3-Hydroxyacyl-ACP dehydrase
cfa (HP0416)	Cyclopropane fatty acyl phospholipid synthase
fabI (HP0195)	Enoyl-ACP reductase I
ufaA (HP0773)	Involved in unsaturated fatty acid biosynthesis

Abbreviation: SAM, *S*-adenosylmethionine

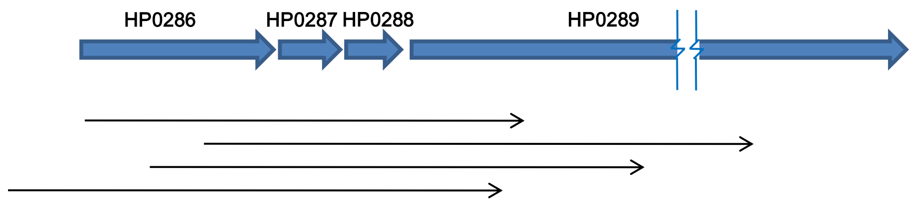

Supplementary Figure 1. The DNA segment common to the complementing clones from the clone bank plasmids. The map at the top of the panel is a section of the pathogenic *H. pylori* strain 26695 genome sequence. HP0287 and HP0288 are ORFs annotated as coding for a hypothetical protein and a membrane protein, respectively. The arrows below the region map represent the four clone bank plasmids that complemented growth the *E. coli* Δ*bioH* mutant strain STL24on biotin-free media.

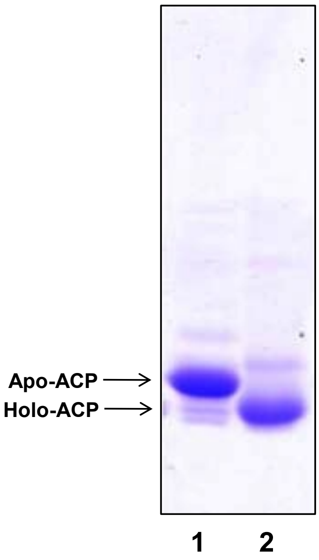

Supplementary Figure 2. Purification of *H. pylori* holo-ACP.

The *H. pylori* apo-ACP was expressed in *E. coli* Rosetta (DE3) pLysS transformed with pBHK531 carrying the *H. pylori acpP* gene (lane 1). The plasmid pBHK532 carrying the *H. pylori acpS* gene was also transformed into the strain to express the holo-ACP (lane 2). The *H. pylori* apo-ACP (lane 1) and holo-ACP (lane 2) were purified as described previously for the *E. coli* proteins [9].

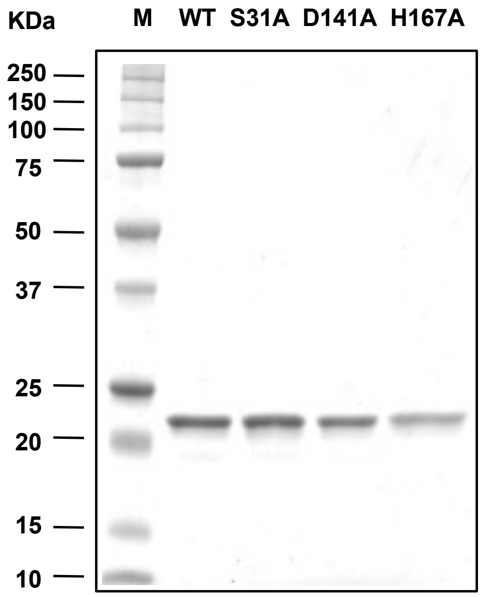

Supplementary Figure 3. SDS-PAGE analysis of the purified BioV and three mutant BioV proteins. Protein samples were separated by electrophoresis using 4-20% gradient SDS-PAGE.

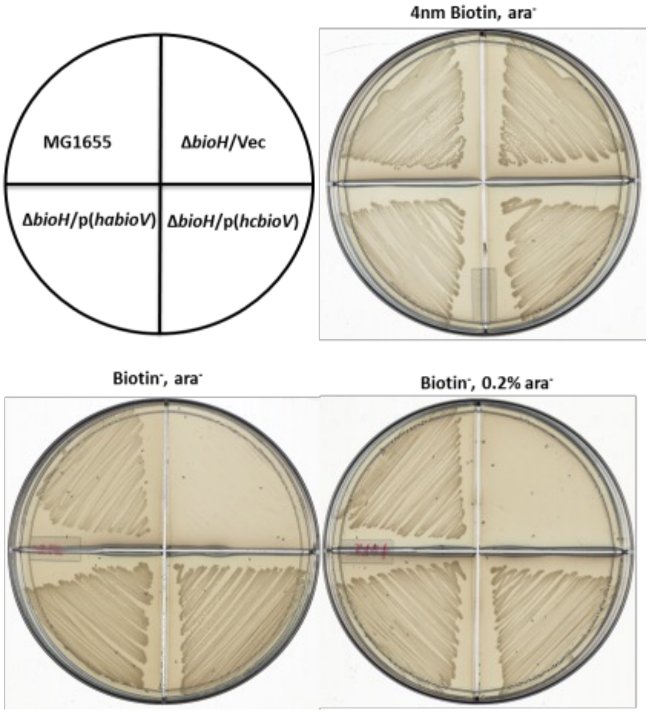

Supplementary Figure 4. Expression of the *bioV* homologues from *Helicobacter acinonychis* or *Helicobacter cetorum* allows growth of an *E. coli* Δ*bioH* strain. Transformants of strain STL24 (an *E. coli ΔbioH* strain) were grown at 37°C on biotin-free minimal medium. Growth was tested in either the presence or the absence of arabinose as in Fig. 2. The growth of all strains in the presence of biotin was used as a positive control. The strains tested were: MG1655 (wild type), STL24 carrying plasmids pZL394 and pZL395 encoding *HcbioV* and *HabioV*, respectively, or the vector plasmid (Vec), pBAD24M.

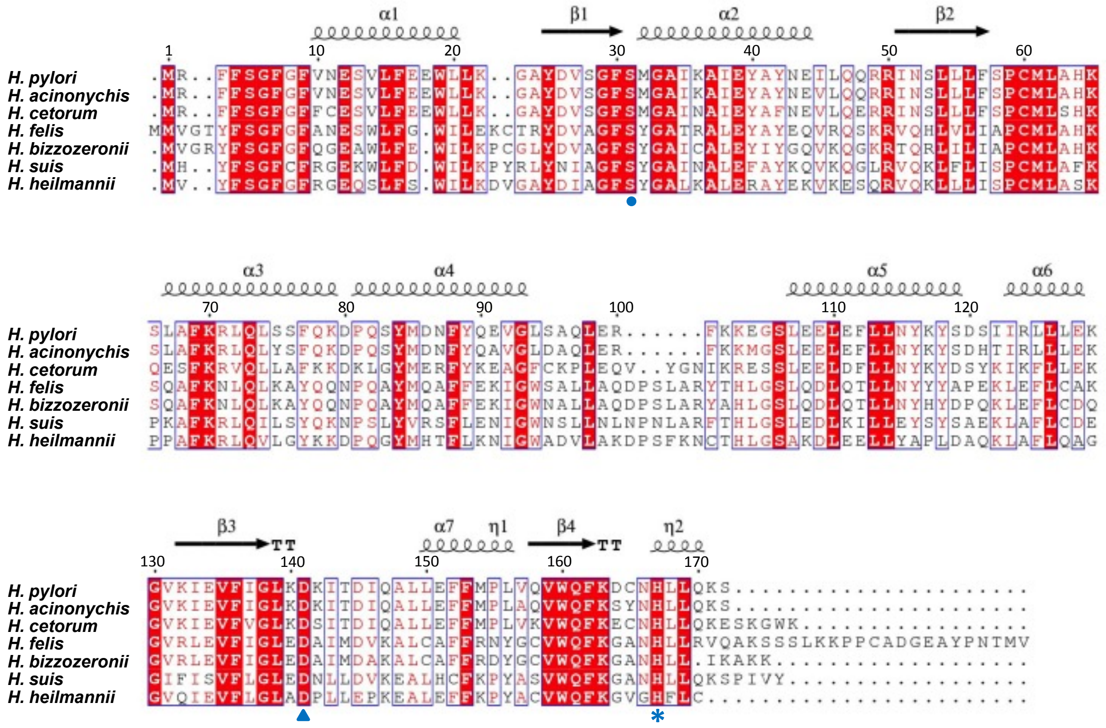

Supplementary Figure 5. Multiple protein sequence alignments of seven BioV homologues from different *Helicobacter* species.

The predicted protein sequences of the BioV homologues are HP0287 from *H. pylori*, Hac_0547 from *H. acinonychis*, HCD_02280 from *H. cetorum*, HFELIS_03760 from *H. felis*, HBZC1_04080 from *H. bizzozeronii*, HSUHS5_1172 from *H. suis* and HHE01_17100 from *H. heilmannii*. The identical residues are in white letters with red background, and the varied residues are in black letters. The predicted protein secondary structure of BioV is given in cartoon form, which is based on the structural architecture of the arylesterase from *Pseudomonas fluorescens* (PDB: 3HI4) [10]. Designations: α: alpha-helix; β: beta-sheet; T: Turn; η: coil. The solid circle, triangle and asterisk denote the active-site residues.

Supplemental References

1. Lin, S., Hanson, R.E. & Cronan, J.E. Biotin synthesis begins by hijacking the fatty acid synthetic pathway. *Nat Chem Biol* **6**, 682-8 (2010).
2. Choi-Rhee, E. & Cronan, J.E. A nucleosidase required for in vivo function of the S-adenosyl-L-methionine radical enzyme, biotin synthase. *Chem Biol* **12**, 589-93 (2005).
3. Choi-Rhee, E. & Cronan, J.E. Biotin synthase is catalytic in vivo, but catalysis engenders destruction of the protein. *Chem Biol* **12**, 461-8 (2005).
4. Tomb, J.F. et al. The complete genome sequence of the gastric pathogen *Helicobacter pylori*. *Nature* **388**, 539-47 (1997).
5. Heuermann, D. & Haas, R. A stable shuttle vector system for efficient genetic complementation of *Helicobacter pylori* strains by transformation and conjugation. *Mol Gen Genet* **257**, 519-28 (1998).
6. O'Sullivan, D.J. & Klaenhammer, T.R. High- and low-copy-number *Lactococcus* shuttle cloning vectors with features for clone screening. *Gene* **137**, 227-31 (1993).
7. Zhu, L., Lin, J., Ma, J., Cronan, J.E. & Wang, H. Triclosan resistance of *Pseudomonas aeruginosa* PAO1 is due to FabV, a triclosan-resistant enoyl-acyl carrier protein reductase. *Antimicrob Agents Chemother* **54**, 689-98 (2010).
8. Guzman, L.M., Belin, D., Carson, M.J. & Beckwith, J. Tight regulation, modulation, and high-level expression by vectors containing the arabinose PBAD promoter. *J Bacteriol* **177**, 4121-30 (1995).
9. Cronan, J.E. & Thomas, J. Bacterial fatty acid synthesis and its relationships with polyketide synthetic pathways. *Methods Enzymol* **459**, 395-433 (2009).
10. Yin de, L.T. et al. Switching catalysis from hydrolysis to perhydrolysis in *Pseudomonas fluorescens* esterase. *Biochemistry* **49**, 1931-42 (2010).
